# *Cyperus esculentus* L. and *Tetracarpidium conophorum* Müll. Arg. Supplemented Diet Improved Testosterone Levels, Modulated Ectonucleotidases and Adenosine Deaminase Activities in Platelets from L-NAME-Stressed Rats

**DOI:** 10.3390/nu13103529

**Published:** 2021-10-08

**Authors:** Ayodeji Augustine Olabiyi, Vera Maria Morsch, Ganiyu Oboh, Maria Rosa Chitolina Schetinger

**Affiliations:** 1 Functional Foods and Nutraceuticals Unit, Department of Medical Biochemistry, Afe Babalola University, P.M.B. 5454, Ado Ekiti 360001, Nigeria; ayoaustin@mail.ufsm.br; 2 Functional Foods and Nutraceuticals Unit, Department of Biochemistry, Federal University of Technology, P.M.B. 704, Akure 340001, Nigeria; goboh2001@yahoo.com; 3 Center of Natural and Exacts Sciences, Department of Biochemistry and Molecular Biology, Federal University of Santa Maria, Santa Maria 97105-900, RS, Brazil; veramorsch@gmail.com

**Keywords:** TN, WN, L-NAME, erectile dysfunction, platelets, androgen, purinergic

## Abstract

In hypertensive individuals, platelet morphology and function have been discovered to be altered, and this has been linked to the development of vascular disease, including erectile dysfunction (ED). The impact of nutritional supplementation with *Cyperus esculentus* (tiger nut, TN) and *Tetracarpidium conophorum* (walnut, WN) on androgen levels, ectonucleotidases, and adenosine deaminase (ADA) activities in platelets from L-NAME (Nω-nitro-L-arginine methyl ester hydrochloride) challenged rats were investigated. We hypothesized that these nuts may show a protective effect on platelets aggregation and possibly enhance the sex hormones, thereby reverting vasoconstriction. *Wistar* rats (male; 250–300 g; *n* = 10) were grouped into seven groups as follows: basal diet control group (I); basal diet/L-NAME/Viagra (5 mg/kg/day) as positive control group (II); ED-induced group (basal diet/L-NAME) (III); diet supplemented processed TN (20%)/L-NAME (IV); diet supplemented raw TN (20%)/L-NAME (V); diet supplemented processed WN (20%)/L-NAME (VI); and diet supplemented raw WN (20%)/L-NAME (VII). The rats were given their regular diet for 2 weeks prior to actually receiving L-NAME (40 mg/kg/day) for ten days to induce hypertension. Platelet androgen levels, ectonucleotidases, and ADA were all measured. L-NAME considerably lowers testosterone levels (54.5 ± 2.2; *p* < 0.05). Supplementing the TN and WN diets revealed improved testosterone levels as compared to the control (306.7 ± 5.7), but luteinizing hormone levels remained unchanged. Compared to control groups, the L-NAME-treated group showed a rise in ATP (127.5%) hydrolysis and ADA (116.7%) activity, and also a decrease in ADP (76%) and AMP (45%) hydrolysis. Both TN and WN supplemented diets resulted in substantial (*p* < 0.05) reversal effects. Enhanced testosterone levels and modulation of the purinergic system in platelets by TN and WN could be one of the mechanisms by which they aid in vasoconstriction control.

## 1. Introduction

Platelets are one of the most essential biological cell fragments in maintaining vascular integrity and facilitating main and secondary haemostasis following vessel injury [1]. Platelet formation and function have been found to be altered in hypertensive people, which may be linked to increased vascular disease [2,3]. In hypertension, enhanced platelet activation and aggregation has also been linked to cardiovascular changes [4]. Other studies [5,6], as well as studies by our group [7,8,9], have employed the nitric oxide synthase (NOS) inhibitor, Nω-nitro-L-arginine methyl ester hydrochloride (L-NAME) as an experimental hypertension model. Due to the emergence and success of sildenafil citrate (Viagra) in 1998, the overall general information available regarding erectile dysfunction (ED) has increased [10,11,12] over the past decades. Viagra (VG) operates by increasing nitric oxide (NO) actions on vascular or corpus cavernosum, thereby causing smooth muscle cell relaxation [10,11,13]. As a result of the therapeutic use of VG, the key role of NO in mediating normal erection and in the aetiology of erectile dysfunction (ED) has been highlighted. However, many side effects have been reported on the use of VG, therefore consumers seeking complementary medicines and/or alternatives has provoked a great interest in research into medicinal plant/food products which are cheaper and safer. ED can have psychological, neurological, hormonal, vascular, cavernosal, or a mixed pathophysiological basis [14]. Furthermore, platelets from hypertensive individuals have been shown to have a higher proclivity for spontaneous aggregation and are extremely hypersensitive to agonists like adenosine diphosphate (ADP) [15,16]. The hydrolysis of extracellular nucleotides is carried out by a multi-enzymatic component on the surface of platelets. This enzyme complex includes the enzymes ectonucleoside triphosphate phosphohydrolase (E-NTPDase), ecto-5′-nucleotidase, and ecto-adenosine deaminase (ADA) [17]. ATP and ADP are converted to AMP [18] by E-NTPDase, while AMP is converted to adenosine by ecto-5′-nucleotidase [19]. The irreversible deamination of adenosine to inosine is catalysed by adenosine deaminase (ADA) [20]. These ecto-enzymes constitute a well-organized enzymatic series that controls the extracellular levels of adenine nucleotides and nucleosides, which are critical in maintaining good haemostasis and thrombogenesis, principally through controlling platelet aggregation [21]. In a number of studies [22,23], androgen levels and substitution have been associated with sexual function, most notably erection quality, desire, and ejaculatory function. Hypogonadism, however, has been linked to ED. According to anatomic studies, androgen receptors are abundantly produced in the male genital tract, the spinal nucleus, the medial preoptic nucleus of the hypothalamus, and the bulbocavernosus muscle [24,25]. 

The tiger nut (TN) tuber is often referred to as Zulu nut, yellow nut grass, ground almond, edible rush, and rush nut, and it belongs to the Cyperaceae grass family. The amino acid arginine [26], which is a contributing factor to nitric oxide bioavailability, has been found to be one of the components of tiger nut tuber. In addition, the nut has been reported to be a natural therapy for treating inflammatory disorders like atherosclerosis [27], preventing heart attacks, and improving blood circulation [28]. Walnut (WN) is a tropical tree in the Euphorbiaceae family [29]. It is widely distributed in Nigeria’s southern region [29]. Walnut nuts have been used locally for decades, and they have several nutritional and therapeutic benefits. Due to the polyphenolics that make up the active ingredients in these nuts, TN and WN supplementation diet has been detected to improve sexual behaviour [30], modulate biochemical indices related to erectile function [31,32], and affect extracellular metabolism of ATP and adenosine via the NOS/cGMP/PKG signalling pathway in the kidney [32]. Our hypothesis in this study is that these nuts will show a protective effect on platelets aggregation and possibly enhance the sex hormones, thereby reverting the effect of hypertension. Therefore, considering ethno-medicinal qualities of TN and WN in the vascular system, as well as the importance of enzymes that degrade adenine nucleotides in the mechanism of thromboregulation, the current study aims to look at the activity of E-NTPDase, ecto-5′-nucleotidase, and ADA in platelets, as well as sex hormone levels from L-NAME (Nω-nitro-L-arginine methyl ester hydrochloride)-stressed rats.

## 2. Materials and Methods

### 2.1. Chemicals and Reagents

Sigma-Aldrich, Inc. (St Louis, MO, USA) provided L-NAME, adenosine, Coomassie brilliant blue G, Trizma base, sodium azide HEPES, nucleotides, and Percoll reagent, while Reagen (Colombo, PR, Brazil) provided bovine serum albumin, K_2_HPO_4_. All of the other chemical substances used in this experiment were of the purest quality, and the water was distilled in glass.

### 2.2. Sample Preparation

TN and WN were purchased from the Nigerian city of Akure in May of 2016. Authentication was conducted at the Department of Biology at the Federal University of Technology in Akure, Nigeria. In order to remove stones and other dirt, the nuts were carefully washed under running water. The edible component of the walnut was separated from the inedible section, and the whole tiger nut was used. Following that, a fraction of the tiger nut was roasted in an electric oven for about thirty minutes at 120 °C (categorized as tiger nut processed), which is the manner in which it is commonly consumed. The raw tiger nut component was dried in an oven to a uniform weight at 45 °C (categorized as raw tiger nut), and the two samples were pulverized, defatted in cold n-hexane, and stored in an airtight container until use. As is conventional, a portion of the walnut was also roasted over an electric gas stove for 45 min at 100 °C (shell removed after cooking). Both the raw (uncooked) and cooked (shell removed) walnut fractions were dried in a vacuum oven to a uniform weight at 45 °C. Additionally, proximate analyses were performed on the nut samples (unpublished data) to establish the nutritional composition of the nuts for feed formulation relative to the amount of protein (Table 1).

### 2.3. Animals

*Wistar* rats (male; 80–100 days; 250–300 g) were used in this experiment. The Central Animal House provided all of the animals (Federal University of Santa Maria, Brazil). The rats were kept at a constant temperature of 23 ± 1 °C with a 12 h dark/light cycle and were given unlimited food and drink. The rats were used in accordance with the rules of the National Council for the Control of Animal Experiments (CONCEA) as well as international standards. All experimental protocols were authorized by the Animal Ethics Committee of the Federal University of Santa Maria, Brazil (Project Ethical Approved number: CEUA 7019030816).

### 2.4. Design of Experiments

The rats were randomly separated into seven groups of ten animals after two weeks of acclimatization (*n* = 10) as follows: basal diet control group (I); basal diet/L-NAME/Viagra (5 mg/kg/day) as positive control group (II); ED-induced group (basal diet/L-NAME) (III); diet supplemented processed TN (20%)/L-NAME (IV); diet supplemented raw TN (20%)/L-NAME (V); diet supplemented processed WN (20%)/L-NAME (VI); and diet supplemented raw WN (20%)/L-NAME (VII). The rats were fed their normal food for two weeks before being given L-NAME to induce vasoconstriction for ten days. Throughout the experiment, daily feed intake was monitored. Gavage route of administration was used to produce ED with L-NAME (40 mg/kg/day) [8,17]. In order to expose the animals in the normal control group (I) to the same stress as the others, they were given water via gavage during the experiment. These rats were euthanized 24 h after the last treatment session, and sex hormone levels (testosterone and luteinizing) were measured using commercially available kits (Randox Laboratories Ltd.—Crumlin, Dublin, Northern Ireland, UK) in accordance with the manufacturer’s instructions. Additionally, relevant biochemical parameters were evaluated and analysed.

### 2.5. Hormonal Assays

Serum testosterone and luteinizing (LH) hormone levels were measured using an enzyme-linked immunosorbent assay (ELISA) kit. The testosterone equine testosterone antigen (ELA) was based on the principle of competitive binding between testosterone in the test specimen and testosterone-horseradish peroxidase (HRP) conjugate for a constant amount of anti-testosterone. For the incubation, anti-testosterone-coated wells were incubated with 25 μL of testosterone standards, control, samples, and 100 μL testosterone-HRP conjugate reagent at room temperature for 60 min. During the incubation, a fixed number of wells were HRP-labelled. Testosterone hydrogen-filled molecular graph (HFG) competes with the endogenous testosterone in the standard, sample, or quality control serum for a fixed number of binding sites of the specific testosterone antibody. Thus, the amount of testosterone peroxidase conjugate immunologically bound to the well progressively decreases as the concentration of testosterone in the specimen increases. Unbound testosterone peroxidase conjugate was then removed, and the wells were washed. Next, a solution of 3,3′,5,5′-tetramethylbenzidine (TMB) reagent was then added and incubated at room temperature for 15 min, resulting in the development of a blue colour. The colour development was stopped with the addition of a stop solution, and the absorbance was measured using a spectrophotometer at 450 nm. Additionally, for the LH hormonal assay, sixteen 12 × 75 mm disposable plastic test tubes were labelled for the standards. Two tubes were used for each sample. Blank reagent (300 μL), standards (200 μL), controls (200 μL), and clinical samples (200 μL) were added to the sample bottles. One hundred microlitres of LH antiserum were added to the tubes and vortexed. The tubes were incubated for 30 min at room temperature. One millilitre of precipitating reagent was added to all sample tubes, and the sample tubes were then vortexed and centrifuged at 1500× *g* for 15 min. The supernatant was carefully decanted from all tubes, except tubes 1 and 2, immediately after centrifugation, inverting the tubes gently to avoid disturbing the precipitate. Then, the supernatant was discarded properly. The radioactivity in the pellets and tubes 1 and 2 was measured in a gamma counter for 1 min to obtain at least 40,000 counts for (57Co) and 75,000 counts for (1251) in the total count tubes. The total counts in tubes 1 and 2 depended upon the efficiency of the scintillation counter in use and the age of the tracer.

### 2.6. Platelet Preparation

The following minor modifications were made to the method used by Lunkes et al. [33] for preparing platelet-rich plasma (PRP). Whole blood was collected using a cardiac puncture and 0.120 M sodium citrate was used as an anticoagulant. The blood–citrate system was centrifuged at 1600× *g* for 15 min. PRP was centrifuged at 14,000× *g* for 30 min before being cleaned twice in 3.5 mM HEPES buffer, pH 7.0, comprising 142 mM NaCl, 2.5 mM KCl, and 5.5 mM glucose. The enzymatic activities of platelet pellets were measured after they were re-suspended in HEPES buffer.

### 2.7. Determination of E-NTPDase and Ecto-5′-Nucleotidase Activities

The platelets were enzymatically tested for E-NTPDase (EC 3.6.1.5) in a reaction medium comprising 5 mM KCl, 1.5 mM CaCl2, 0.1 mM EDTA, 10 mM glucose, 225 mM sucrose, and 45 mM Tris–HCl buffer, pH8.0, as described in a previous study [34]. The reaction mixture was added to 20 microliters of enzyme preparation (8–12 g protein) and incubated for 10 min at 37 °C. In either case, the reaction was started by adding 1.0 mM ATP or ADP to a final concentration of 1.0 mM and incubating for 20 min. In a reaction medium containing 10 mM MgSO4 and 100 mM Tris–HCl buffer, pH7.5, in a final volume of 200 µL, the activity of 5’-nucleotidase (EC 3.1.3.5) was evaluated following the method of Heymann et al. [35]. The reaction mixture was pre-incubated at 37 °C for 10 min with 20 microliters of enzyme preparation (8–12 g of protein). The reaction was started by adding AMP to a final concentration of 2.0 mM, and it took 20 min to complete. In both cases, the reaction was stopped by adding 200 µL of 10% trichloroacetic acid to achieve a final concentration of 5%. The tubes were then refrigerated for 10 min on ice. This method was followed by Chan et al. [36], who used malachite green as a colorimetric reagent and KH2 PO4 as a standard to evaluate the amount of liberated inorganic phosphate (Pi). The synaptosomal fraction was added after the trichloroacetic acid addition to adjust for non-enzymatic nucleotide hydrolysis. Enzyme activities were measured in nanomoles of Pi released per minute per milligram of protein for each sample examined in triplicate.

### 2.8. Adenosine Deaminase Activity Determination

Guisti and Galanti [37] established a method for determining ADA activity that is based on the reliable detection of ammonia production when ADA acts in excess of adenosine. In brief, 50 µL of enzyme preparation was incubated with 21 mmol/L of adenosine at pH 6.5 for 60 min at 37 °C. In the experiment, the protein concentration was set to be between 0.7 and 0.9 mg/mL. Units per litre (U/L) were used to calculate the results. One unit (1 U) of ADA is defined as the quantity of enzyme necessary to release 1 mmol of ammonia per minute from adenosine under normal test conditions.

### 2.9. Protein Determination

Bradford’s Coomassie blue technique [38] and serum albumin as a standard were used to determine protein levels.

### 2.10. Statistical Evaluations

One-way ANOVA was performed first, followed by Tukey’s multiple range tests; a significant difference was defined as *p* < 0.05 in both analyses. All data were provided as a mean ± standard error of mean (SEM). The statistical studies were performed using the GraphPad Prism 5 software tool.

## 3. Results

### 3.1. Feed Intake Measurement and the Impact of TN and WN Supplementation in L-NAME-Stressed Rats

After gavage administration of L-NAME (40 mg/kg/day), no significant (*p* > 0.05) changes in body weight or feed intake were found among the test groups (data not published).

### 3.2. Impact of TN and WN Supplementation on the Activity of EC 3.6.1.5, EC 3.1.3.5, and ADA in the Platelets of L-NAME-Stressed Rats

**Figure 1:**
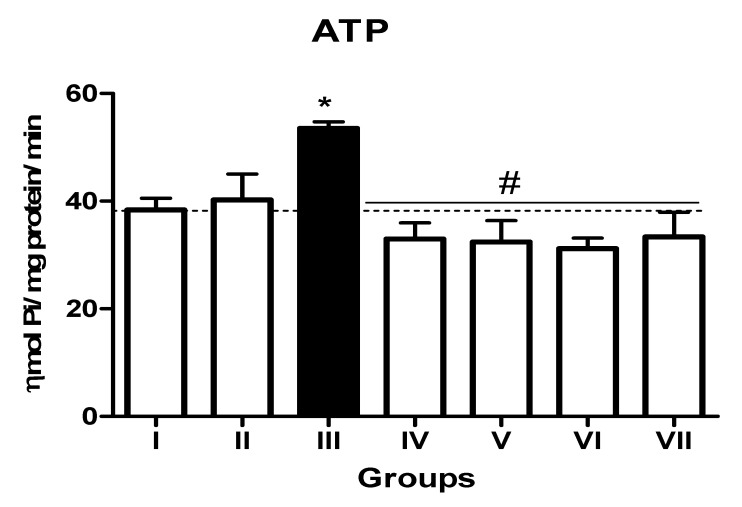
E-NTPDase (ATP as substrate) activity in platelets of L-NAME-stressed rats treated with tiger nut and walnut supplemented diets. Data are presented as mean ± SEM (*n* = 10). The bars with various symbols are statistically different (*p* < 0.05). * represents a significant difference from the control (I) group. # represents a significant difference from the ED (III) group (one-way ANOVA followed by post hoc Tukey, *, # denote *p* < 0.05, Key: Group I: Normal control placed on a basal diet; Group II: Positive control placed on a basal diet/L-NAME/Viagra (5 mg/kg/day); Group III: ED-induced group placed on a basal diet/L-NAME (40 mg/kg/day); Group IV: Processed TN supplemented diet (20%)/L-NAME; Group V: Raw TN supplemented diet (20%)/L-NAME (20%); Group VI: Processed WN supplemented diet (20%)/L-NAME; Group VII: Raw WN supplemented diet (20%)/L-NAME.

**Figure 2:**
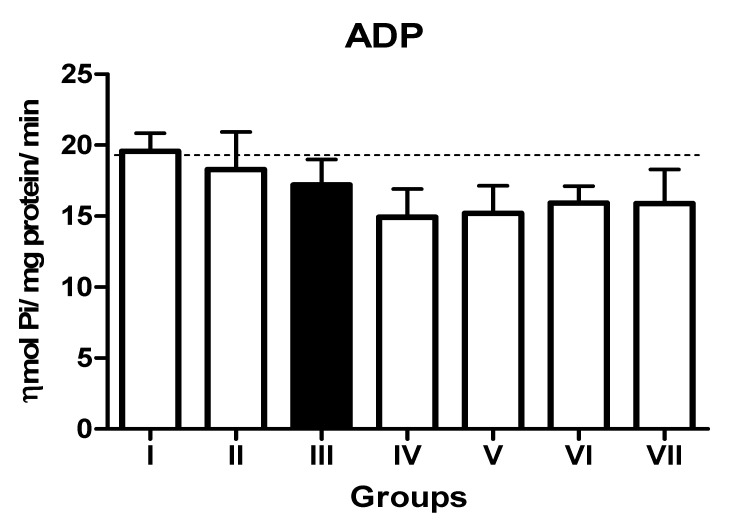
NTPDase (ADP as substrate) activity in platelets of L-NAME-stressed rats treated with tiger nut and walnut supplemented diets. Data are presented as mean ± SEM (*n* = 10). The bars with various symbols are statistically different (*p* < 0.05). Key: Group I: Normal control placed on a basal diet; Group II: Positive control placed on a basal diet/L-NAME/Viagra (5 mg/kg/day); Group III: ED-induced group placed on a basal diet/L-NAME (40 mg/kg/day); Group IV: Processed TN supplemented diet (20%)/L-NAME; Group V: Raw TN supplemented diet (20%)/L-NAME (20%); Group VI: Processed WN supplemented diet (20%)/L-NAME; Group VII: Raw WN supplemented diet (20%)/L-NAME.

**Figure 3:**
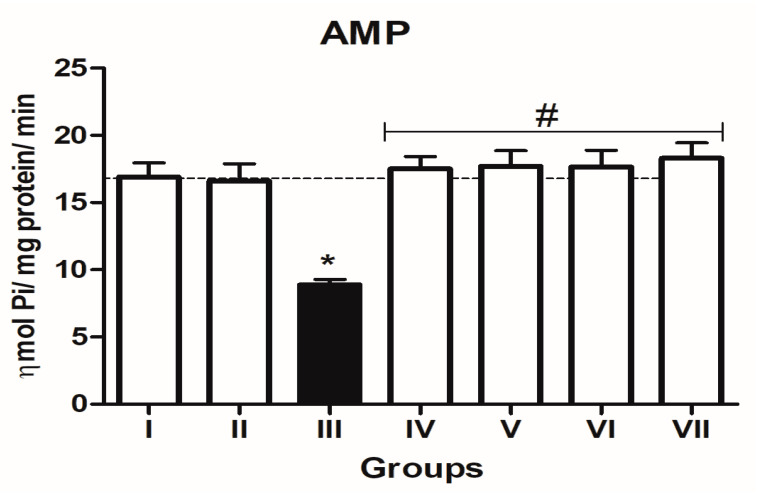
5′-nucleotidase (AMP as substrate) activity in platelets of L-NAME-stressed rats treated with tiger nut and walnut supplemented diets. Data are presented as mean ± SEM (*n* = 10). The bars with various symbols are statistically different (*p* < 0.05). * represents a significant difference from the control (I) group. # represents a significant difference from the ED (III) group (one-way ANOVA followed by post hoc Tukey, *, # denote *p* < 0.05; Key: Group I: Normal control placed on a basal diet; Group II: Positive control placed on a basal diet/L-NAME/Viagra (5 mg/kg/day); Group III: ED-induced group placed on a basal diet/L-NAME (40 mg/kg/day); Group IV: Processed TN supplemented diet (20%)/L-NAME; Group V: Raw TN supplemented diet (20%)/L-NAME (20%); Group VI: Processed WN supplemented diet (20%)/L-NAME; Group VII: Raw WN supplemented diet (20%)/L-NAME.

**Figure 4:**
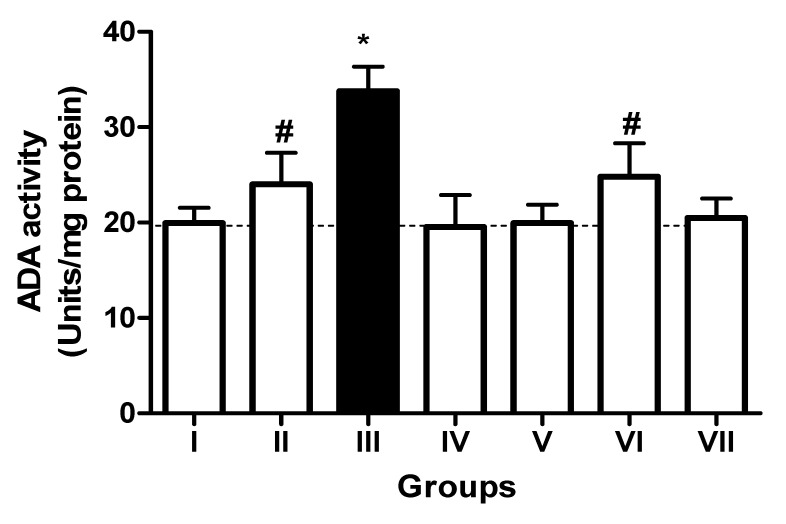
Adenosine deaminase (ADA) activity in platelets of L-NAME-stressed rats treated with tiger nut and walnut supplemented diets. Data are presented as mean ± SEM (*n* = 10). The bars with various symbols are statistically different (*p* < 0.05). * represents a significant difference from the control (I) group. # represents a significant difference from the ED (III) group (one-way ANOVA followed by post hoc Tukey, *, # denote *p* < 0.05; Key: Group I: Normal control placed on a basal diet; Group II: Positive control placed on a basal diet/L-NAME/Viagra (5 mg/kg/day); Group III: ED-induced group placed on a basal diet/L-NAME (40 mg/kg/day); Group IV: Processed TN supplemented diet (20%)/L-NAME; Group V: Raw TN supplemented diet (20%)/L-NAME (20%); Group VI: Processed WN supplemented diet (20%)/L-NAME; Group VII: Raw WN supplemented diet (20%)/L-NAME.

In the L-NAME-treated group, the results for E-NTPDase (EC 3.6.1.5; ATP and ADP, as substrates) and ecto-5′-nucleotidase (EC 3.1.3.5) activities in platelets revealed an increase in ATP (127.5%; *p* < 0.01) hydrolysis with a reduction in ADP (72%; *p* < 0.05) and AMP (68%; *p* < 0.05) hydrolysis when compared to the normal and positive control groups. However, it is interesting to note that both TN (raw = 78%; processed = 80%) and WN (raw = 79.8%; processed = 75%) supplemented diets caused a decrease in E-NTPDase using ATP as substrate (F(4,49) = 2.840; *p* < 0.01). Moreover, a non-significant increase in the hydrolysis of platelet’s ADP (F(4,49) = 0.8351; *p* < 0.05) for TN (raw = 68%; processed = 66.4%) and WN (raw = 69.1%; processed = 67.3%) and a significant decrease in AMP (F(4,49) = 4.759; *p* < 0.05], for TN (raw = 67%; processed = 65%) and WN (raw = 66%; processed = 68%) were observed. (Figure 1, Figure 2 and Figure 3). Furthermore, the L-NAME-treated group (116.7%) had higher ADA activity (*p* < 0.001) than the control (66.7%) group, whereas the TN (raw = 67%; processed = 66.8%) and WN (raw = 69.3%; processed = 71.2%) supplemented diet caused a significant (*p* < 0.05) reduction in ADA activity (F(4,49) = 3.835; *p* < 0.001) compared to controls (Figure 4).

### 3.3. Impact of TN and WN Supplementation on Testosterone and Luteinizing Hormone (LH) Levels in L-NAME-Stressed Rats

Result from this study showed that L-NAME reduces testosterone levels (54.5 ± 2.2) significantly (*p* < 0.05). However, supplementation with TN (raw = 118.2 ± 9.3; processed = 88.7 ± 0.7) and WN (raw = 123.8 ± 5.7; processed = 145 ± 6.0) was shown to increase testosterone levels in the serum as compared to the control (306.7 ± 5.7), while LH levels were not altered across the groups, as shown in Table 2.

## 4. Discussion

This study’s hypothesis was accepted owing to the observed results. The pathophysiology of several disorders, including strokes, venous thrombosis, arterial thrombosis, and myocardial infarction, has been linked to platelet dysfunction [39]. The release of vasoconstrictor substances and reactive oxygen species by platelet and leucocyte adhesion has been demonstrated to have a negative impact on erection, which may play a role in the progression of ED [40]. In hypertensive patients, the aetiopathogenesis of ED is complex, influenced by a range of psychological and biological factors. Among the organic causes are diabetic neuropathy, oxidative stress, dyslipidaemia, arterial hypertension, endothelial dysfunction, hypogonadism, and pharmaceutical side effects [41,42]. Platelets are also thought to have a function in atherothrombosis pathogenesis [43]. In comparison to L-NAME-treated and normal control rats, results obtained from the activity of E-NTPDase demonstrated a modulatory influence of TN and WN on platelet activity. Hypertension, according to Lunkes et al. [44], is a contributing factor for thrombus development. E-NTPDase activity in blood platelets has been found to change in a variety of physiological and pathological situations, implying that it is involved in thromboregulation [45,46]. As a result, it is believed that both TN and WN contain polyphenolics [9,31], which aid in thromboregulation. Our findings, which revealed that L-NAME increased adenosine deaminase activity, while TN and WN reversed this effect when compared to the control, also demonstrated the influence of adenosine. This suggests that ADA’s decrease with TN and WN supplementation will improve adenosine bioavailability, which is consistent with studies showing that adenosine prevented platelet aggregation by activating the A2A receptor on platelets and caused vasodilation in smooth muscle cells via the A2B receptor [47,48]. Nitric oxide is a vasodilator that inhibits platelet activity by increasing cyclic guanosine monophosphate (cGMP) levels and thereby restricting vasoconstriction [49,50]. Nitric oxide may affect platelet responses by stimulating soluble guanylate cyclase, which catalyses the production of the secondary messenger cGMP [51]. Platelet cGMP levels rise, causing a change in intracellular Ca^2+^ mobilization that favours smooth muscle relaxation [52,53,54]. Both adenosine and NO have been found to be powerful vasodilators and have been linked to erectile dysfunction [9,13]. We determined that L-NAME induced a drop in ADA activity across groups, which is consistent with what we found in our lab. TN and WN, on the other hand, were able to counteract the effects of L-NAME. Thousands of research papers on testosterone and its biological consequences have been published lately, with more than a third of them published in the last decade. One important factor fuelling this growing scientific interest is testosterone’s impact on human health, particularly in men. Not only is it a basic regulator of male reproduction and the maturation of external genital features [55], but testosterone has also been related to men’s well-being and overall health [56]. According to our findings in this experiment, L-NAME induced a reduction in serum testosterone levels, which agrees with the findings of numerous reports in which low testosterone levels were linked to diabetes [57,58], Alzheimer’s [59,60], atherosclerosis [61,62], cancer [63], osteoporosis [64,65], and infertility [66,67], which are common characteristics of aging disorders/diseases. However, no toxicological effect of the supplemented diet was observed in the experimented rats. The mechanism by which testosterone secretion increases without parallel increase in the secretion of LH is still unknown. Therefore, further efforts are ongoing to determine if there is a direct effect of TN and WN on Leydig cells which bypasses the hypothalamic-pituitary (HP) axis.

## 5. Conclusions

According to our findings, TN and WN improved testosterone levels and modulated the activities of ectonucleotidases in platelets of L-NAME-stressed rats as a model of hypertension. As a result, testosterone boosting and the modulation of the E-NTPDase as well as ADA in the pathogenesis of hypertension may represent mechanisms by which TN and WN contribute to erectile function. However, the mechanism of action by which testosterone secretion increased without a corresponding increase in the secretion of LH is still being investigated. The potential attributes of these nuts can be said to be provided by the presence of bioactive components (polyphenolics) in TN and WN which has been documented from our laboratory [9,32].

## Figures and Tables

**Table 1:** Feed formulation chat for basal, tiger nut (TN) and walnut (WN) dietary supplementation for both control and test groups.

	Groups						
Materials	I	II	III	IV	V	VI	VII
Skimmed milk	37.5	37.5	37.5	33.1	33.1	21.3	26.3
Oil	10.0	10.0	10.0	10.0	10.0	10.0	10.0
Vitamin mix.	4.0	4.0	4.0	4.0	4.0	4.0	4.0
Corn Starch	48.5	48.5	48.5	32.9	32.9	44.7	39.7
Tig 1	-	-	-	20.0	-	-	-
Tig 2	-	-	-	-	20.0	-	-
Wal 1	-	-	-	-	-	20.0	-
Wal 2	-	-	-	-	-	-	20.0
VIAGRA	-	+	-	-	-	-	-
L-NAME	-	+	+	+	+	+	+
Total (g)	100	100	100	100	100	100	100

Wal 1: processed walnut; Wal 2: raw walnut, Tig 1: processed tiger nut; Tig 2: raw tiger nut. Skimmed milk contains 32% protein. 1 g vitamin mixture Vitamin A: 3200 IU, 600 IU Vitamin D3, 2.8 mg vitamin E, 0.6 mg vitamin K3, 0.8 mg vitamin B1, 1 mg vitamin B2, 6 mg niacin, 2.2 mg pantothenic acid, 0.8 mg vitamin B6, 0.004 mg vitamin B12, 0.2 mg folic acid, 0.1 mg biotin H2, 70 mg choline chloride, 0.08 mg cobalt, 1.2 mg copper, 0.4 mg iodine, 8.4 mg iron, 16 mg manganese, 0.08 mg selenium, 12.4 mg zinc, and 0.5 mg antioxidant. Group I: Normal control placed on a basal diet; Group II: Positive control placed on a basal diet/L-NAME/Viagra (5 mg/kg/day); Group III: ED-induced group placed on a basal diet/L-NAME (40 mg/kg/day); Group IV: Processed TN supplemented diet (20%)/L-NAME; Group V: Raw TN supplemented diet (20%)/L-NAME (20%); Group VI: Processed WN supplemented diet (20%)/L-NAME; Group VII: Raw WN supplemented diet (20%)/L-NAME.

**Table 2:** Testosterone and luteinizing hormone levels from L-NAME-stressed rats treated with TN and WN supplemented diets.

Groups	Testosterone Hormone	Luteinizing Hormone
	(ng/dL)	(mIU/mL)
I	306.7 ± 5.7 ^a^	28.5 ± 0.1 ^a^
II	102.4 ± 7.4 ^d^	27.8 ± 0.9 ^a^
III	54.5 ± 2.2 ^f^	27.1 ± 0.2
IV	88.7 ± 0.7 ^e^	27.5 ± 0.6 ^a^
V	118.2 ± 9.3 ^c^	29.2 ± 0.7 ^a^
VI	145.5 ± 6.0 ^b^	29.8 ± 0.4 ^a^
VII	123.8 ± 5.7 ^c^	29.5 ± 0.3 ^a^

The results are presented (mean ± SD) in triplicates. Different letters indicate a significant difference taken into account by Tukey post hoc test at *p* < 0.05. Group I: Normal control placed on a basal diet; Group II: Positive control placed on a basal diet/L-NAME/Viagra (5 mg/kg/day); Group III: ED-induced group placed on a basal diet/L-NAME (40 mg/kg/day); Group IV: Processed TN supplemented diet (20%)/L-NAME; Group V: Raw TN supplemented diet (20%)/L-NAME (20%); Group VI: Processed WN supplemented diet (20%)/L-NAME; Group VII: Raw WN supplemented diet (20%)/L-NAME.

## Abbreviations

ATP	adenosine triphosphate
ADP	adenosine diphosphate
AMP	adenosine monophosphate
ADA	adenosine deaminase
E-NTPdase	ectonucleotidases
ED	erectile dysfunction
L-NAME	Nω-nitro-L-arginine methyl ester hydrochloride
NOS	nitric oxide synthase
SIL	sildenafil
TN	tiger nut
WN	walnut
